# How Will Video Conference Fatigue Affect Participants of MICE in the With-COVID-19 Era? Focusing on Video Conference Quality, Social Presence Theory, and Flow

**DOI:** 10.3390/ijerph19084601

**Published:** 2022-04-11

**Authors:** Mi-Hwa Jang, Eui-Yul Choi

**Affiliations:** 1 Department of Industry-University Cooperation, Keimyung University, Daegu 42601, Korea; pw332@daum.net; 2 Department of Marine Sport Sciences, Korea Maritime & Ocean University, Busan 49112, Korea

**Keywords:** mental health, MICE, COVID-19, video conference fatigue, video conference quality, social presence, flow

## Abstract

Is our mental health at risk due to spending a significant amount of time online due to the COVID-19 pandemic? In the new era that we are living in, where we live a life that coexists with the virus, we are participating in video conferences held online rather than on-site in order to slow the spread of the virus. Video conferencing has become our necessity since March 2020, and is becoming a new standard, especially in the MICE industry. Recently, however, people who have excessively used video conference platforms are complaining of video conference fatigue, which is a new negative emotion such as stress, anxiety, and worry as well as general work fatigue. Therefore, this study focused on the mechanism of video conferencing in MICE, which is rapidly digitally converted by the virus, and the digital psychological factors of the participants. This study derived the quality attributes of video conferencing in MICE and empirically analyzed the relationship with digital psychological factors of the video conference participants, such as video conference fatigue, social presence, and flow. One hundred and thirty-eight valid questionnaires collected from participants of several international academic conferences held in EXCO, Daegu, Korea, from 23 to 28 May 2021, were analyzed. The main results are as follows. First, unlike general video conference fatigue, MICE video conference fatigue was not found to be related to the preceding and following variables. This is due to the characteristics of the MICE video conference and the expertise of the participants. Second, social presence was identified as an important variable in MICE video conferencing. Although media-mediated, the feeling of being present with the presenter and participants was found to affect the participants’ flow in the video conference. Third, in this study, the fun factor was identified as the most important video conference quality that can enhance the social presence of the video conference participants of MICEs.

## 1. Introduction

MICE, as an abbreviation of Meeting, Incentive Tour, Convention, and Exhibition, collectively refers to events where a large number of people meet for the purpose of business. The March 2020 pandemic changed the MICE industry, limiting the movement and gathering of people. Public health guidelines such as quarantine, social distancing and social contact closure have been in effect to all countries around the world [1]. In the early days of the COVID-19 outbreak, most MICEs around the world were canceled or postponed, such as the Mobile World Congress 2020 (MWC 2020), which was canceled with great losses [2]. At that time, the use of video conferencing tools did not spread to the general public and there was a fear of ignorance of the virus. In the new ‘normal’ era, however, where we live a life that coexists with the virus, we are safely participating in online or hybrid MICE in our own shelter. As such, video conferencing has grown in importance and has become another standard during the pandemic [3]. In addition, video conferencing is increasing new potential value for the MICE industry as an alternative to the on-site meeting format that could not be held due to the COVID-19 pandemic [4].

It is also expected that the hosting of MICEs in virtual space will continue for some time as it is predicted that our lives and people’s actions will not return to pre-pandemic standards after the end of the virus [5]. Virtual MICE technology was developed a long time ago but has not been widely used. After the pandemic, especially in the field of MICE, the technology related to the video conferencing platform developed, and its use increased rapidly. As such, the rapid digital transformation of the meeting field in the MICE industry caused by the virus has raised the need for research related to the mechanism of video conferencing and the digital psychological factors of the participants. So far, however, there have been few academic studies related to this. Therefore, this study intended to make an empirical attempt to analyze not only video conference quality but also various psychological factors of video conference participants of MICE, such as social presence, fatigue, and flow.

The study had the following detailed objectives: (1) to derive video conference quality that reflects the characteristics of video conferencing and to suggest its constituent dimensions; (2) to apply the social presence, which is again attracting attention due to the rapid increase in communication mediated by media, and to suggest its constituent dimensions; (3) to conceptualize video conference fatigue and flow of MICE participants; (4) to examine the relationship between video conference quality and the digital psychological factors of the participants, such as social presence, fatigue, and flow. As such, this study aimed to understand the psychological factors of video conference participants of MICEs and, furthermore, to suggest academic and practical implications for deriving incentives for more people to participate in virtual MICE conferences classified as essential economic activities, even in the global pandemic.

## 2. Literature Review and Research Question Development

### 2.1. Video Conference Quality

Video conferencing is a meeting that proceeds where seeing and listening with each other while transmitting images and voices is through a network from afar [6]. Video conferencing held using representative tools, such as Hangout Teams, Webex, or Zoom, is characterized by real-time communication through the network and immediate communication between the moderator and participants and between participants themselves [7]. In the era of coexistence with COVID-19, it is expected that for the time being, the number of video conferences, or hybrid conferences combined with virtual and on-site meetings, will increase [5].

However, since it has not been long since video conferencing has changed from a substitute to a necessity, there are few studies that have tried to derive a conceptual definition and attributes of video conferencing, especially in the MICE industry. Lee, Yoon, and Yoon [8] presented 25 attributes of 6 factors, including technical, physical, participant experience, online conference service, program planning, and event management, as factors to consider when planning a video conference. Among them, they identified novel content planning, technical problem solving, and the stable use of video solutions as important items. In addition, Hwang-Bo and Kim [9] simply analyzed the service experience of customers using Zoom and Teams, which are the most used tools so far in the study of video conferencing platform service user experience. As such, since there are not many approaches to define the concept of video conferencing and derive its attributes, WEBQUAL 4.0, including Usefulness, Information, Interaction, and Security, which is based on the technical characteristics of video conferencing held through media and the web, has been measured as the quality of video conferencing [8,10,11]. In addition, in this study, a fun factor identified to induce the user’s active reaction in the web service and affect their attitude, satisfaction, and behavior [12,13] was added.

In the relationship between video conference quality and related psychological variables, it was found that the quality provided through media-mediated communication, such as video conference, remote education, and online shopping, which has increased rapidly, affects social presence [14,15,16]. Social presence is a digital psychological factor that should be continuously noted even after digital transformation is expected to accelerate. Meanwhile, several studies found that the technical quality of video conferencing can reduce video conference fatigue, which has been newly introduced after the March 2020 pandemic [17,18,19,20]. Additionally, Cho and Lim [15] and Kwon, Kim, and Chang [21] found that the interaction quality of real-time internet broadcasting has a positive effect on viewers’ flow. Based on the previous studies that have been cited above, therefore, we set out the following research question.

Research Question 1: Does video conference quality affect social presence, fatigue, and flow among participants of MICEs?

### 2.2. Social Presence Theory

Under the pandemic, the presence theory has been reemphasized with the rapid increase of communication through media such as remote education and video conferencing [22,23,24]. The presence is the feeling of actually experiencing the mediated experience [25,26,27,28,29], and it is divided into telepresence and social presence. Telepresence means forgetting the media’s mediation and feeling as if users are actually there [28,30,31] and social presence refers to the feeling of being with other persons in a media-mediated environment [25,27,32,33,34].

In particular, social presence is attracting attention as a factor that affects the user’s psychologically and behaviorally, such as a mediated attitude toward others, reality illusion, learning, memory, and mental health [25,27]. Additionally, in the video conference field of the MICE industry, where interaction with other participants is important, the feeling of being with other people can be an important psychological variable for participants. Therefore, this study intended to pay attention to social presence in order to find out whether video conferencing through media can be as effective as a face-to-face conference. Social presence was first studied focusing on mediated media by Short, Williams, and Christie [35], and they defined it as salience with others in communicative interactions. They were criticized for a limitation in not reflecting people’s psychological connectivity because they focused only on the existence of others [25,27,36]. Therefore, social presence should be measured on how people feel and perceive psychological connections with others in mediated interactions [25,27,32,37,38].

In the relationship between social presence and other psychological variables, a study by Knox [39] found that the lower the social presence, the greater the feeling of Zoom fatigue, also called video conference fatigue. On the contrary, an empirical study on the antecedents and consequences of SNS users’ fatigue found that the higher the SNS social presence, the more influential the SNS fatigue [40]. Meanwhile, social presence has long been attracting attention as an important influencing variable on flow in media-mediated communication research. In studies on e-learning and Internet broadcasting [15,41,42,43], social presence was found to positively affect flow. Based on the above previous studies, therefore, we set out the following research question.

Research Question 2: Does social presence affect video conference fatigue and flow among participants of MICEs?

### 2.3. Video Conference Fatigue

After the pandemic, video conferencing is not a substitute but a necessity [1]. It has enabled us to be able to work in our shelter-in-place [17]. As the usage of video conferencing tools has increased dramatically in recent years, the daily use of Zoom, a representative video conferencing application, has increased from approximately 10 million in December 2019 to 200 million in March 2020 and 300 million in April 2020 [44,45]. As such, video conferencing has spread as a new type of meeting in the business and education fields as well as in the MICE industry.

The advantages of video conferencing are clear in maintaining social distance and replacing face-to-face meetings during the pandemic. Some people adapt well to a new media platform, for most people, however, it can be a huge challenge [46]. In order to adapt to new technologies, we inevitably face various problems, such as machine malfunctions, difficulties in the network, and increased Internet traffic [47]. People who have recently used video conferences have felt new negative emotions such as stress, exhaustion, tiredness or worrying through the platforms, as well as general work fatigue [47]. Accordingly, several researchers have proposed this fatigue as a new concept called “Zoom Fatigue” [1] or “Video Conference Fatigue” [48]. In this study, besides Zoom, more platforms are expected to be developed in the near future, so the feeling of exhaustion from participating in video conferencing is defined as video conference fatigue.

In recent research, Bennett et al. [48] showed that video conferencing during the pandemic was associated with more fatigue accumulation than daily fatigue. In addition, Spataro [49] suggested that video conferencing is more fatiguing than face-to-face conferencing due to the continuous increase in concentration. Video conference fatigue is a terminology that appeared in 2020 and there is not enough research on how to measure the fatigue or the relationship with other variables. This study is premised on the results that fatigue felt during the media platform use affects the user’s other psychological aspects [50,51], especially flow [52,53]. Therefore, we set out the following research question based on the above previous studies.

Research Question 3: Does video conference fatigue affect participants’ flow among participants of MICEs?

### 2.4. Video Conference Flow

Flow is a concept originally started by cultural anthropologists in the study of rituals [54]. Flow was defined as the transitional stage between a former and a later status in a person’s rite of passage. During this period, humans feel disconnected from reality, confused about their identity, and feel as if time has stopped. After this, the social psychologist Csikszentmihalyi [55], defined flow as a state of being psychologically immersed or completely immersed in an experience or object. Additionally, Csikszentmihalyi [56] explained it as an optimal experience in which an individual is completely immersed in an object and defined it as a state in which interest and curiosity synergized with each other, making it impossible to recognize the passage of time. This psychological state acts as an important factor in maintaining people’s positive psychology toward a certain object [57].

Recently, the experience of flow has also been shown in the interaction mediated by media, such as human and computer, and found as an important variable affecting the performance in media-mediated communication, such as remote education and video conferencing, which have surged due to COVID-19 [58,59,60,61,62,63,64,65]. Therefore, in the video conference field of MICEs, a systematic approach is needed to examine the antecedent variables affecting participants’ flow.

## 3. Methodology

### 3.1. Theoretical Framework

**Figure 1:**
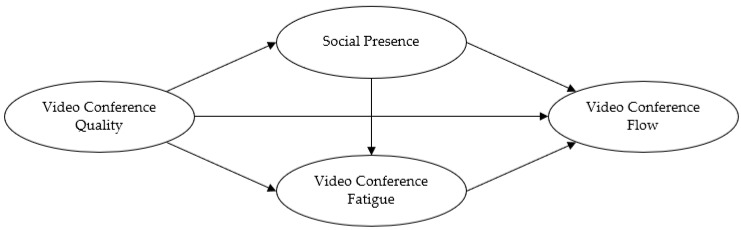
Theoretical Framework on the relationship between video conference quality and psychological factors of video conference participants of MICEs; social presence, fatigue, and flow.

This study aimed to derive factors for expanding participants to virtual MICE conferencing, by studying the relationship between video conference quality and psychological factors of video conference participants of MICEs, such as social presence, fatigue, and flow. In order to accomplish the main purposes of the study, we set out the following theoretical framework, which we based on the previous studies that have been cited above (Figure 1):

### 3.2. Subject of Survey

A survey for participants of several international academic conferences held in EXCO (Daegu Exhibition and Convention Center), the Daegu metropolitan city, South Korea, was conducted to analyze the relationship between video conference quality, social presence, fatigue, and flow. Two pre-trained surveyors surveyed conference participants who had recently participated in a video conference in the MICE industry. The surveyors stayed at EXCO for four days from May 23 to 28, 2021, and distributed questionnaires to the participants who voluntarily expressed their intention to participate. They explained to the participants the purpose and importance of the survey, and anonymity and confidentiality were ensured in completing the survey. A total of 180 survey questionnaires were distributed, 152 copies were collected, and 138 copies were used for analysis, excluding invalid questionnaires such as insincere responses. Of the 138 participants, 83 were men (60.1%) and 55 were women (39.9%). By age, 73 of the participants were in their twenties (52.9%), 27 were thirties (19.6%), 21 were forties (15.2%), and 17 were over fifty (12.3%). Looking at the number of video conferences participated in, 65 participants were in the 1–2 times category (47.1%), 36 in the 3–5 times category (26.1%), and 37 in the 6 times or more category (26.8%).

### 3.3. Measures

Video conference quality was derived from 5 factors and 27 items based on previous studies [8,10,11,12,13] (See Table 1). The items of the quality of video conferencing were validated and verified by three MICE experts to secure the appropriateness and representativeness of the questionnaire. They were measured on a 5-point Likert scale, ranging from 1 (*strongly disagree*) to 5 (*strongly agree*).

In this study, the social presence of video conferencing was defined as the psychological involvement in social interaction as a continuous process in which participants recognize the presence of the presenter and other participants (See Table 2). As such, two factors and sixteen items were adapted from [27,37,66,67] with some modifications. They were validated and verified by three MICE experts to secure the appropriateness and representativeness of the questionnaire. The items of the social presence of video conferencing were measured on a 5-point Likert scale, ranging from 1 (*strongly disagree*) to 5 (*strongly agree*).

In order to measure video conference fatigue, the items used in the study of [1] were modified and supplemented to fit the purpose of this study (See Table 3). Fourteen items were validated and verified by three MICE experts to secure the appropriateness and representativeness of the questionnaire. The items of the fatigue of video conferencing were measured on a 5-point Likert scale, ranging from 1 (*strongly disagree*) to 5 (*strongly agree*).

Video conference flow is defined as the optimal experience in which participants are completely immersed in the video conference. Based on previous studies [68,69,70], sixteen items were validated and verified by three MICE experts to secure the appropriateness and representativeness of the questionnaire (See Table 4). The items of the flow of video conferencing were measured on a 5-point Likert scale, ranging from 1 (*strongly disagree*) to 5 (*strongly agree*).

### 3.4. Data Processing

The questionnaire data collected for this study was analyzed using the SPSS 26.0 statistical program as follows. Exploratory factor analysis was conducted to derive the factors of the quality and social presence of video conferencing. Cronbach’s alpha coefficient was estimated to verify the reliability of measuring tools for video conference quality, social presence, fatigue, and flow. Additionally, correlation values between all the variables were estimated using the Pearson correlation coefficient to identify their relationships. After this process, descriptive statistics for all the variables were computed. Finally, multiple regression analyses were performed to predict the influence relationship between the variables. All the statistical verifications were based on a significance level of 0.05.

## 4. Results

### 4.1. Reliability and Validity

Exploratory factor analyses on video conference quality (27 items) and social presence (16 items) were performed using principal component analysis with Varimax rotation method. Seven items that hindered unidimensionality or did not satisfy the factor loading of 0.5 or higher were dropped. The quality of video conference consisted of five factors, namely, usefulness, information, interaction, security, and fun. Social presence consisted of two factors, presenter social presence and participant social presence. Each factor with the eigenvalue greater than 1 contained 3 to 8 items. For video conference quality, Table 5 showed that five factors explained 72.117% of total variance and a total of 22 items were converged. Cronbach’s α was located between 0.838 and 0.915 to meet the criteria (higher than 0.7) that Nunnally and Bernstein [71] pointed out as having high internal consistency, thereby ensuring data reliability. For social presence, Table 6 showed that two factors explained 68.467% of total variance and a total of 14 items were converged. Cronbach’s α was located between 0.875 and 0.945, indicating that data were found to have high internal consistency. Additionally, as a single factor, fatigue and flow regarding video conference, data were found to have sufficient reliability according to the Cronbach’s α of 0.888 and 0.832, respectively.

### 4.2. Descriptive Statistics and Correlation Analysis

As shown in Table 7, the conference participants did not show a clear difference in video conference quality, but the highest mean value was shown by information (M = 3.547, SD = 0.530) and it was followed by interaction (M = 3.199, SD = 0.726), usefulness (M = 3.192, SD = 0.736), security (M = 2.964, SD = 0.821), and fun (M = 2.810, SD = 0.809). Presenter social presence (M = 2.764, SD = 0.715) was found to be stronger than participant social presence (M = 2.459, SD = 0.764). In addition, video conference quality, social presence, and flow all show a positive (+) correlation.

### 4.3. Effect of Video Conference Quality on Social Presence, Fatigue, and Flow

Multiple regression analysis was conducted to explain the effect of video conference quality on social presence, fatigue, and flow. As shown in the results presented in Table 8, among the factors in video conference quality, fun, interaction, and security positively predicted presenter social presence, where fun was found to be greater than the other significant factors in the relative importance on presenter social presence. In regard to explanatory power, the model accounted for 39.0% of the variance in presenter social presence. VIFs (Variance Inflation Factors) ranged from 1.340 to 1.434, respectively, which indicated that multicollinearity did not exist among the independent variables [72]. Second, fun and usefulness showed positive effects on participant social presence. Fun was found to be greater than usefulness in the relative importance of participant social presence. The variance in participant social presence explained by the model was 40.4%. Third, the results for all the factors of the quality were statistically nonsignificant for video conference fatigue. The model accounted for 7.6% of the variance in video conference fatigue. Last, only the fun factor positively predicted video conference flow, whereas the results for information, interaction, usefulness, and security were statistically nonsignificant. The variance in the flow explained by the model was 43.1%.

### 4.4. Effect of Social Presence on Video Conference Fatigue and Flow

Multiple regression analysis was used to predict video conference fatigue and flow with social presence. As shown in the results presented in Table 9, the results for all the factors of social presence were statistically nonsignificant for video conference fatigue. The variance in the flow explained by the model was 0.2%. On the other hand, both presenter and participant social presence positively predicted video conference flow, where presenter social presence was found to be greater than the participant in the relative importance on the flow. The variance in the flow explained by the model was 59.8%.

### 4.5. Effect of Video Conference Fatigue on Video Conference Flow

Multiple regression analysis was conducted to explain the effect of video conference fatigue on the flow. As shown in Table 10, video conference fatigue was found to be statistically nonsignificant. The variance in video conference flow explained by the model was 1.6%.

## 5. Discussion

Video conferencing has become a necessity for people after the COVID-19 pandemic of March 2020 [1] and is increasing its new potential as another standard in the MICE industry [4]. However, the rapid digital transformation of the meeting industry caused by the virus has raised the need for research on the mechanism of video conferencing and the digital psychological factors of participants. This study tried to confirm the quality attributes of video conferencing and to empirically analyze the relationship with various the digital psychological factors of video conference participants, such as video conference fatigue, social presence, and flow. Accordingly, based on the results of the study, the main implications are presented as follows.

First of all, in the MICE industry, video conference fatigue was not affected by video conference quality and social presence and was not identified as an important variable such as not affecting flow. In the two years since the March 2020 pandemic, as many meetings, including general business meetings, have mostly been converted to online, several researchers [1,48] have paid attention to video conference fatigue as a new concept. They argued that during the pandemic, video conferencing accumulated more fatigue than routine fatigue, and Spataro [49] also suggested that video conferencing is more tiring than on-site conferencing due to the continuous increase in concentration. Such fatigue was found to be influential on various psychological variables [52,53]. In this study, however, it was found that the fatigue of video conferencing in MICEs was not related to the preceding and subsequent variables. This is because video conferencing in MICEs is a professional one run by a meeting planner, and related services such as the quality of video conference and operation are good, so participants are not expected to feel very tired. Additionally, it is estimated that video conference participants of the MICE industry have more voluntary motives than those who participate in general business conferences, and they do not have any major problems with the technical part of using video conferencing.

On the other hand, in this study, social presence was identified as an important variable in video conferencing of MICEs. Both presenter social presence and participant social presence were found to be influential on video conference flow. Social presence has been emphasized as influencing flow and performance, especially in studies related to remote education and livestreaming broadcasting [15,41,42,43]. In particular, Swan and Shih [67] found that social presence for presenters had a greater effect on learning satisfaction and perceived learning than that of peer learners. Similarly, in this study, it was found that presenter social presence had a greater effect on flow than that of other participants. As such, social presence was identified as the most important psychological variable in MICE video conferencing, which is used as an essential commodity due to the special situation of the epidemic. Therefore, MICE officials need to consider factors that can enhance social presence among participants when planning and operating video conferences.

In this study, among the factors of video conference quality, fun was found to be the most influential factor that can enhance social presence of the video conference. Most of the participants in MICE video conferences are voluntary, and they have different characteristics from general conference participants, such as networking, escaping from everyday life, and gathering information. Therefore, it is considered that meeting planners should pay attention to the fun factor that can give participants a feeling that the overall process of participating in a video conference is enjoyable and free from ordinary life.

## 6. Conclusions

Unlike general video conference fatigue, MICE video conference fatigue was not found to be related to the preceding and following variables. On the other hand, social presence was identified as an important variable in MICE video conferences. The feeling of being present with the presenter and participants was found to affect participants’ flow in video conferences. Lastly, the fun factor was identified as the most important video conference quality that can enhance the social presence of video conference participants of the MICE industry.

Although this study has great significance in focusing on the digital psychological factors of video conference participants in MICEs, it leaves the possibility of various follow-up studies due to its limitations as an a priori study. First, there are insufficient data due to limitations in accessing research subjects due to the corona virus. At the time of the data collection period of this study, most meetings in MICEs were canceled or postponed due to the second stage of social distancing in Korea. Only a few meetings that were ready to be held as a video conference were held online or as a hybrid (online + offline). Video conferences, which are held entirely online only, make it difficult to access participants, and the total number of participants in hybrid conferences was not large. Therefore, it is necessary to expand the number of participants who have experience in video conferences, which are expected to be continuously used even two years after the pandemic in March 2020 and even after the end of the virus. Second, demographic characteristics may be biased because of the targeting of specific conference participants. Therefore, it will be necessary to expand the research data by targeting participants of conferences in various fields. Third, MICE video conferencing has different characteristics from general business video conferencing in terms of purpose, attributes, and motivation for participation. Therefore, it is necessary to expand a study faithful to the essence of MICE video conferencing by utilizing various related variables.

## Figures and Tables

**Table 1:** Video conference quality.

Factor	Item	Source
Usefulness	Using the video conference was easy Using the video conference was convenient Using the video conference gave me a feeling I could do it The overall layout of the video conference was attractive The overall design of the video conference was suitable for the characteristics of the conference The overall design of the video conference was excellent	Lee, Yoon, Yoon (2021); Barnes, Vidgen (2002); Xu, Choi (2018)
Information	The video conference provided accurate information The video conference provided reliable information The video conference provided timely information The video conference provided information relevant to me The video conference information was easy to understand The video conference delivered information in an appropriate format	
Interaction	Overall communication was good in the video conference Communication with presenter was good in the video conference Communication with another participant was good in the video conference Communication with the moderator (service company) was good in the video conference I felt a sense of belonging through video conference participation I felt a sense of community through video conference participation	
Security	Participation in the video conference was safe in terms of personal information management The video conference was secure Personal information will never be leaked through the video conference A safe payment system was operated for personal security in video conferences	
Fun	I had a fun participating in the video conference I enjoyed participating in the video conference I was excited to participate in the video conference I didn’t know the time was passing when I participated in the video conference I felt like I was out of my routine when I participated in the video conference	Huang (2003); Muehling, Sprott, Sprott (2004)

**Table 2:** Video conference social presence.

Factor	Item	Source
Presenter Social Presence	I felt like I was in the same room with the presenter I felt like I was actually with the presenter I felt like the presenter was presenting in front of me I understood exactly what the presenter was saying as if I was listening to it in person I felt emotionally connected to the presenter I felt close to the presenter I thought the presenter gave me an immediate reaction I felt psychologically close to the presenter	Hwang (2007); Nowak (2001); Kwon (2011); Swan, Shih (2005)
Participant Social Presence	I felt like I was in the same room with other participants I felt like I was actually with other participants I felt like other participants were in front of me I understood exactly as if I was listening to a conversation with other participants I felt emotionally connected with other participants I felt close to other participants I thought other participants gave me an immediate reaction I felt psychologically close to other participants	

**Table 3:** Video conference fatigue.

Factor	Item	Source
Video Conference Fatigue	I felt tired I felt exhausted I felt mentally drained My vision got blurred My eyes felt irritated I experienced pain around my eyes I wanted to avoid social situations I just wanted to be alone I needed time by myself I didn’t feel like doing anything I often felt too tired to do other things I felt emotionally drained I felt irritable I felt moody	Fauville, Luo, Muller Queiroz, Bailenson, Hancock (2021)

**Table 4:** Video conference flow.

Factor	Item	Source
Video Conference Flow	I had fun participating in the video conference I was interested in participating the video conference I enjoyed participating in the video conference I had a good time participating in the video conference I was excited to participate in the video conference I was relaxed while participating in the video conference I didn’t think about other things while participating in the video conference I was not aware of distractions and obstacles while participating in the video conference I didn’t know what was going on around me while participating in the video conference I was completely immersed or absorbed while participating in the video conference I focused on my interests while participating in the video conference I felt like time flew by very quickly while participating in a video conference I didn’t recognize that time went by so quickly while participating in the video conference I felt like time was changing while participating in the video conference I felt like time stopped while participating in the video conference I completely forgot myself while participating in the video conference I didn’t care what other people thought of me while participating in the video conference I wasn’t worried about the outcome while participating in the video conference I wasn’t interested in how I was expressing myself while participating in the video conference	Guo, Xiao, Van Toorn, Lai, Seo (2016); Kaur, Dhir, Chen, Rajala (2016); Novak, Hoffman, Yung (2000)

**Table 5:** Exploratory factor analysis and reliability for video conference quality.

Factor	Item	Factor Loading					Cronbach’s α
Fun	VCQ23	**0.906**	0.180	0.093	0.081	0.055	0.915
	VCQ26	**0.831**	0.313	0.080	0.193	0.083	
	VCQ24	**0.821**	0.315	0.106	0.190	0.123	
	VCQ25	**0.793**	0.047	0.125	0.000	0.117	
	VCQ27	**0.755**	0.089	0.101	0.171	0.212	
Information	VCQ8	0.127	**0.758**	0.009	0.051	0.138	0.838
	VCQ9	0.147	**0.725**	0.080	0.127	0.097	
	VCQ7	0.165	**0.710**	0.174	0.194	0.033	
	VCQ10	0.068	**0.696**	0.220	0.118	0.185	
	VCQ11	0.179	**0.681**	0.177	0.227	0.044	
	VCQ12	0.125	**0.652**	0.090	0.041	0.105	
Interaction	VCQ13	0.086	0.176	**0.906**	0.111	0.088	0.906
	VCQ15	0.187	0.098	**0.873**	0.127	0.032	
	VCQ16	0.063	0.195	**0.844**	0.105	0.152	
	VCQ14	0.130	0.159	**0.723**	0.303	0.190	
Usefulness	VCQ1	0.125	0.265	0.156	**0.820**	0.094	0.889
	VCQ4	0.139	0.105	0.178	**0.818**	0.140	
	VCQ5	0.164	0.073	0.144	**0.816**	0.188	
	VCQ3	0.082	0.216	0.105	**0.798**	0.136	
Security	VCQ19	0.061	0.189	0.130	0.169	**0.862**	0.862
	VCQ21	0.162	0.160	0.163	0.194	**0.829**	
	VCQ22	0.278	0.031	0.100	0.149	**0.795**	
Eigenvalue		3.742	3.518	3.137	3.097	2.371	
Variance (%)		17.011	15.991	14.258	14.079	10.778	
		Cumulative Variance (%) = 72.117					
		Kaiser–Meyer–Olkin Measure of Sampling Adequacy = 0.847					
		Bartlett‘s Test of Sphericity: χ^2^ = 2084.541, df = 231, *p* < 0.01					

VCQ = video conference quality; Factor loadings of 0.5 or higher are in bold.

**Table 6:** Exploratory factor analysis and reliability for social presence.

Factor		Item	Factor Loading		Cronbach’s α
Participant Social Presence		SP13	**0.885**	0.261	0.945
		SP14	**0.874**	0.282	
		SP16	**0.857**	0.282	
		SP10	**0.784**	0.335	
		SP9	**0.747**	0.367	
		SP12	**0.723**	0.418	
		SP15	**0.645**	0.493	
		SP11	**0.635**	0.437	
Presenter Social Presence		SP2	0.306	**0.833**	0.875
		SP3	0.324	**0.798**	
		SP1	0.305	**0.779**	
		SP4	0.297	**0.708**	
		SP5	0.221	**0.643**	
		SP7	0.354	**0.546**	
Eigenvalue			5.351	4.235	
Variance (%)			38.218	30.249	
	Cumulative Variance (%) = 68.467				
	Kaiser–Meyer–Olkin Measure of Sampling Adequacy = 0.909				
	Bartlett‘s Test of Sphericity: χ^2^ = 1683.688, df = 91, *p* < 0.01				

SP = social presence; Factor loadings of 0.5 or higher are in bold.

**Table 7:** Correlation coefficients between latent variables.

Variable	Fun	Information	Interaction	Usefulness	Security	Participant	Presenter	Fatigue	Flow
Fun									
Information	0.429 **								
Interaction	0.319 **	0.387 **							
Usefulness	0.359 **	0.408 **	0.406 **						
Security	0.378 **	0.322 **	0.347 **	0.408 **					
Participant	0.596 **	0.346 **	0.328 **	0.403 **	0.328 **				
Presenter	0.523 **	0.423 **	0.407 **	0.319 **	0.426 **	0.728 **			
Fatigue	−0.205 *	−0.245 **	−0.090	−0.135	−0.149	0.035	0.010		
Flow	0.625 **	0.377 **	0.341 **	0.324 **	0.377 **	0.563 **	0.548 **	−0.125	
*M*	2.810	3.547	3.199	3.192	2.964	2.459	2.764	2.702	2.765
*SD*	0.809	0.530	0.726	0.736	0.821	0.764	0.715	0.705	0.492

* *p* < 0.05, ** *p* < 0.01.

**Table 8:** Results of multiple regression using video conference quality to predict social presence, fatigue, and flow.

DV	IV	*B*	*SE*	ß	*t*	*p*	VIF
Presenter Social Presence *R*^2^ = 0.390 *F* = 16.872 ***	Fun	0.292	0.070	0.330	4.148	0.000 ***	1.372
	Information	0.209	0.109	0.155	1.914	0.058	1.420
	Interaction	0.178	0.077	0.181	2.295	0.023 *	1.340
	Usefulness	−0.015	0.079	−0.015	−0.187	0.852	1.434
	Security	0.170	0.069	0.195	2.473	0.015 *	1.342
Participant Social Presence *R*^2^ = 0.404 *F* = 17.930 ***	Fun	0.456	0.074	0.483	6.140	0.000 ***	1.372
	Information	0.036	0.115	0.025	0.312	0.755	1.420
	Interaction	0.086	0.082	0.082	1.048	0.296	1.340
	Usefulness	0.177	0.084	0.170	2.117	0.036 *	1.434
	Security	0.036	0.072	0.039	0.501	0.617	1.342
Video Conference Fatigue *R*^2^ = 0.076 *F* = 2.165	Fun	−0.097	0.085	−0.112	−1.139	0.257	1.372
	Information	−0.255	0.133	−0.192	−1.922	0.057	1.420
	Interaction	0.043	0.094	0.044	0.453	0.651	1.340
	Usefulness	−0.012	0.096	−0.012	−0.122	0.903	1.434
	Security	−0.047	0.083	−0.055	−0.569	0.571	1.342
Video Conference Flow *R*^2^ = 0.431 *F* = 20.006 ***	Fun	0.310	0.047	0.509	6.620	0.000 ***	1.372
	Information	0.068	0.073	0.073	0.930	0.354	1.420
	Interaction	0.068	0.052	0.101	1.330	0.186	1.340
	Usefulness	0.015	0.053	0.023	0.290	0.773	1.434
	Security	0.070	0.046	0.116	1.530	0.128	1.342

* *p* < 0.05, *** *p* < 0.001.

**Table 9:** Results of multiple regression using social presence to predict video conference fatigue and flow.

DV	IV	*B*	*SE*	ß	*t*	*p*	VIF
Fatigue *R*^2^ = 0.002 *F* = 0.119	Presenter SP	−0.033	0.124	−0.033	−0.265	0.791	2.127
	Participant SP	0.055	0.116	0.059	0.474	0.637	2.127
Flow *R*^2^ = 0.598 *F* = 37.518 ***	Presenter SP	0.225	0.065	0.349	3.466	0.000 ***	2.127
	Participant SP	0.202	0.069	0.294	2.920	0.004 **	2.127

** *p* < 0.01, *** *p* < 0.001.

**Table 10:** Results of multiple regression using video conference fatigue to predict video conference flow.

DV	IV	*B*	*SE*	ß	*t*	*p*
Flow *R*^2^ = 0.016 *F* = 2.175	Video Conference Fatigue	−0.088	0.059	−0.125	−1.475	0.143

## Data Availability

The data presented in this study are available on request from the corresponding author.
